# Innate immune response in COVID-19: single-cell multi-omics profile of NK lymphocytes in a clinical case series

**DOI:** 10.1186/s12964-024-01867-5

**Published:** 2024-10-15

**Authors:** Silvia Barbon, Fabrizio Armellin, Verena Passerini, Sergio De Angeli, Simona Primerano, Laura Del Pup, Elisabetta Durante, Veronica Macchi, Raffaele De Caro, Pier Paolo Parnigotto, Arianna Veronesi, Andrea Porzionato

**Affiliations:** 1 Section of Human Anatomy, Department of Neuroscience, University of Padova, Via Gabelli 65, 35121 Padova, Italy; 2 Foundation for Biology and Regenerative Medicine, Tissue Engineering and Signaling - T.E.S. Onlus, Padova, Italy; 3 Complex Operative Unit of Transfusion Medicine - Marca Trevigiana Local Unit of Health and Social Services 2, Treviso Hospital, Piazzale dell’Ospedale 1, 31100 Treviso, Italy

**Keywords:** COVID-19, Natural killer lymphocytes, Innate immune response, Single-cell multi-omics analysis, BD Rhapsody system

## Abstract

**Background:**

COVID-19 pandemic caused by the Severe Acute Respiratory Syndrome-Coronavirus-2 (SARS-CoV-2) represents the biggest global health emergency in recent decades. The host immune response to SARS-CoV-2 seems to play a key role in disease pathogenesis and clinical manifestations, with Natural Killer (NK) lymphocytes being among the targets of virus-induced regulation.

**Methods:**

This study performed a single-cell multi-omics analysis of transcripts and proteins of NK lymphocytes in COVID-19 patients, for the characterization of the innate immunological response to infection. NK cells were isolated from peripheral blood samples collected from adult subjects divided into 3 study groups: (1) non-infected subjects (*Naïve group*, *n* = 3), (2) post COVID-19 convalescent subjects (*Healed group*, *n* = 3) and (3) patients that were vaccinated against SARS-CoV-2 (*Vaccine group*, *n* = 3). Cells were then analysed by the BD Rhapsody System for the single-cell multi-omics investigation of transcriptome and membrane proteins.

**Results:**

The bioinformatic analysis identified 5 cell clusters which differentially expressed gene/protein markers, defining NK cell subsets as “Active NK cells” and “Mature NK cells”. Calculating the relative proportion of each cluster within patient groups, more than 40% of the *Naïve group* cell population was found to belong to Mature NKs, whereas more than 75% of the *Vaccine group* cell population belonged to the cluster of Active NKs. Regarding the *Healed group*, it seemed to show intermediate phenotype between Active and Mature NK cells. Differential expression of specific genes, proteins and signaling pathways was detected comparing the profile of the 3 experimental groups, revealing a more activated NK cell phenotype in vaccinated patients versus recovered individuals.

**Conclusions:**

The present study detected differential expression of NK cell markers in relation to SARS-CoV-2 infection and vaccine administration, suggesting the possibility to identify key molecular targets for clinical-diagnostic use of the individual response to viral infection and/or re-infection.

**Supplementary Information:**

The online version contains supplementary material available at 10.1186/s12964-024-01867-5.

## Background

Coronavirus disease 2019 (COVID-19), determined by infection with severe acute respiratory syndrome coronavirus 2 (SARS-CoV-2), emerged in late 2019 causing a global pandemic with almost unprecedented morbidity and mortality [1, 2]. Being declared as a global health emergency by the World Health Organization (WHO) [3], COVID-19 continues to be object of intensive clinical research efforts trying to identify the pathogenesis, the immunological implications and the eventual treatment options of this novel disease [4].

Like the previously identified strains SARS-CoV and MERS-CoV, the pathogen SARS-CoV-2 has been found to act in humans by targeting the Angiotensin Converting Enzyme 2 (ACE2) receptor to enter the host cells [5], causing an immunocompromised status (i.e., lymphocytopenia and monocyte abnormalities) [6] and leading to pulmonary infection of different extent. Indeed, the severity of COVID-19 clinical manifestations ranges from asymptomatic or mild upper respiratory symptoms to serious viral pneumonia causing acute respiratory failure [1, 7]. The host reaction to infection and disease progression depends on multiple factors, such as age, sex, concomitant chronic conditions/pathologies (e.g., hypertension, obesity, diabetes, heart failure) and, as already demonstrated, misdirected or imbalanced immune responses by the patient [8].

Despite most studies focused on the implication of T and B lymphocyte response in COVID-19 patients or in their activation after vaccination against SARS-CoV-2, the innate immune response to infection is raising growing interest among clinicians and researchers as the first line of organism defense and possible key factor for decipher mechanisms of host response and disease pathogenesis [9]. Furthermore, natural immunological memory could play an essential role in controlling SARS-CoV-2 infection because it assures cross-protection towards different pathogens. Innate immune cells include macrophages, monocytes, dendritic cells, neutrophils and innate lymphoid cells (ILCs) such as Natural Killer (NK) cells. This lymphocyte subset possesses a number of cell surface, endosomal and cytosolic pattern recognition receptors (PRRs) that identify pathogen-associated molecular patterns (PAMPs) to trigger inflammatory signaling pathways and induce programmed cell death that limit viral infection and promote clearance [10].

Among innate immune cells, NK lymphocytes are responsible for immunosurveillance against viral pathogens and cancer [11]. NK cell role in COVID-19 has become the focus of interest since the first onset of SARS-CoV-2 infection, with clinical data being in constant and rapid increase in the literature (reviewed for example by Deng and colleagues [1]). However, the understanding of genetic and immunophenotypic dysregulation in NK lymphocytes from COVID-19 patients still deserves to be improved. Based on that, this prospective clinical study presents the broad characterization of the innate immune response in the post-infection phase of COVID-19 patients by a single-cell multi-omics approach using the BD Rhapsody™ System technology. To this end, SARS-CoV-2 negative subjects and patients convalescing from COVID-19 who did or did not receive vaccination were enrolled at the Department of Transfusion Medicine of Treviso Hospital, Italy for peripheral blood collection, NK lymphocyte extraction and assessment of cell genetic and immunophenotype profile. The study aim is to define specific indicators of the innate immunological memory which could contribute to the protection from SARS-CoV-2 infection and COVID-19 disease immunopathogenesis.

## Methods

### Ethics statement

Blood samples and patient information were collected after obtaining written and signed informed consent from all participants. The study was approved by the Ethics Committee for the Clinical Trial of Treviso and Belluno Hospitals (DDG n. 1218, 23 July 2020; DDG n. 1276, 30 July 2020).

### Patients

Study participants were enrolled at the Intercompany Department of Transfusion Medicine (DIMT) of Treviso Hospital, in the period between March, 3rd 2021 and March, 29th 2021. Peripheral blood samples for NK cell extraction were collected from a total of 9 volunteer adult subjects (mean age: 48.1 ± 9.7 years; age range: 27–62 years) divided into 3 study groups: (1) non-infected subjects (*Naïve group*, *n* = 3), that were subjects resulting negative to the molecular biology test for SARS-CoV-2 and to the serological test for specific IgG and IgM; (2) post COVID-19 adult convalescent subjects or subjects previously affected by SARS-CoV-2 infection and declared clinically recovered according to the definition contained in the New Coronavirus Regional Procedure (SARS-CoV-2, Rev. 02 of 6.03. 2020) and in the document of the Superior Health Council of 28.02.2020 [12] (*Healed group*, *n* = 3); (3) patients that were vaccinated against SARS-CoV-2 (*Vaccine group*, *n* = 3).

Patients/donors were required to meet the eligibility criteria for plasma donors, in accordance with current legislation. In particular, participants in the study underwent:

- suitability visit (according to DM 2/11/2015);
- blood chemistry tests (blood count, PT, PTT, fibrinogen, total and fractionated protein, AST, ALT, serology for the qualitative detection of anti-SARS-CoV-2 IgG and IgM with lateral flow chromatographic immunoassay rapid test, anti-SARS-CoV-2 antibody titer, NAT-test for HIV, HBV, HCV, HAV, HEV, parvovirus B19, HIV serology, HBV, HCV, Lue serology, TCI and TCD, blood group, lymphocyte subpopulations);
- biomolecular investigations (DNA extraction, mRNA extraction, transcriptome analysis);
- microbiological investigations (oral-nose-pharyngeal swab execution).

### Study design

To investigate COVID-19-induced alterations of the NK lymphocyte population, single cell immunoprofiling of NK cells was performed by combining targeted scRNA-seq with single cell proteomics (BD Rhapsody Abseq). To this end, NK cells were isolated from the three different groups of patients which were previously described (paragraph 2.2) using magnetic beads enrichment, and then analysed by a multi-omics approach by the BD Rhapsody™ System (Becton Dickinson - BD, Franklin Lakes, NJ, USA).

### NK cell isolation

#### Isolation of the peripheral blood mononuclear cell fraction

Peripheral blood samples collected from patients were used in part (3 ml) to perform (a) basal blood count, (b) basal cytofluorimetric morphological examination and (c) definition of HLA profile, according to the routine protocols adopted by the DIMT of Treviso Hospital.

In parallel, 6 ml of each sample were processed by centrifugation at 250 × g for 5 minutes (min) at room temperature (RT) to remove platelets and then diluted 1:1 with Phosphate Buffer Saline (PBS). After that, diluted blood was gently stratified on an equal volume of Ficoll-Histopaque 1077 (Merck LifeScience, Darmstadt, Germany) obtaining two separate phases. Centrifuging samples at 2,500 rpm for 20 min, the Peripheral Blood Mononuclear Cell (PBMC) fraction including lympho-monocyte populations formed a ring between the plasma and Ficoll. The lympho-monocyte ring was then retrieved with a glass Pasteur pipette, washed with 5–10 ml of PBS and centrifuged at 1,500 rpm for 10 min, for 3 times. At the third wash, the cell suspension was filtered with a cell-strainer (70 μm nylon mesh).

#### Enrichment of NK cell populations

NK cell populations were purified from the PBMC fraction of healthy, convalescent and vaccinated subjects by means of the BD IMag™ Human NK Cell Enrichment Set – DM and BD IMag™ Cell Separation Magnet (BD Biosciences, Franklin Lakes, NJ, USA), following the Manufacturer’s instructions. This method allows for the negative selection of NK cells from peripheral blood, using an enrichment cocktail of biotinylated Human antibodies against erythrocytes, platelets, and peripheral leukocytes that are not NK cells. After labeling, magnetic streptavidin-conjugated nanoparticles are added to the cell suspension and bind the cells marked with the biotinylated antibodies. Placing cells within the magnetic field, labeled elements migrate toward the magnet (positive fraction), whereas the unlabeled cells in suspension can be retained (enriched fraction). This negative selection aims to avoid the inadvertent activation of the enriched NK cells by using reagents that do not directly bind to them. To achieve high cell purity, the negative selection of NK cells was performed twice.

After cell enrichment, samples underwent counting and viability tests, and effective enrichment was assessed by flow-cytometry.

Finally, aliquots of NK cells from each patient were frozen at – 80 °C into freezing medium consisting of Fetal Calf Serum (FCS) and 10% Dimethyl sulfoxide (DMSO) (Merck LifeScience) until subsequent multi-omics analysis.

### Multi-omics analysis of NK cell samples by BD Rhapsody™

#### Cell preparation and cytofluorimetric assessment

Cryopreserved NK populations were thawed at 37 °C and transferred drop by drop into the washing medium consisting of RPMI-1640 Medium + 20% Citrate-dextrose solution (ACD) (Merck LifeScience). After centrifugation at 1,500 rpm for 10 min, cell pellet was resuspended in RPMI-1640 Medium + 10% FCS and cell suspension underwent cell count and viability assessment, as well as flow-cytometry evaluation. All primary antibodies used for flow-cytometry assessment were purchased by Becton Dickinson (BD) or BioLegend (San Diego, CA, USA) and are listed in Additional file 1.

#### Single cell labelling with the BD™ single-cell multiplexing kit and BD™ AbSeq

Following flow-cytometry analysis, NK cells from each subject were labeled with the BD™ Human Single-Cell Multiplexing Kit (Cat. No. 633781) and BD™ AbSeq according to the Manufacturer’s protocol. Briefly, 500,000 cells/sample were resuspended in 200 µl BD Stain Buffer (Cat. No. 554656), added to Sample Tag tubes and incubated at RT for 20 min. Samples were then washed 3 times by the addition of 2 ml BD Pharmingen Stain Buffer and centrifugated at 400 × g for 5 min. Cell pellets were resuspended in 500 µl of cold BD Sample Buffer from the BD Rhapsody Cartridge Reagent Kit (Cat. No. 633731). Viability staining and cell counting were performed according to Manufacturer’s indications, by resuspending cells in cold BD Sample Buffer (Cat. No. 650000062) and then labeling them with 2 mM Calcein AM and 0.3 mM Draq7 (Thermo Fisher Scientific, Waltham, MA, USA).

Following cell counting with the BD Rhapsody™ Scanner, 30,000 cells/sample were pooled together to proceed with cell labeling by BD AbSeq. First of all, the pooled cell suspension was incubated for 10 min at RT into BD Pharmingen Human BD Fc Block (Cat. No. 564219) in order to avoid antibody binding to non-specific sites. After that, 100 µl pooled cell suspension labelled with Sample Tags were combined with 100 µl 2X BD AbSeq labelling master mix and incubated on ice for 30 min. Table 1 shows the list of AbSeq (BD Bioscience) used to label NK cells.

Following incubation with AbSeq antibodies, cells were first washed 3 times with cold BD Sample Buffer (BD Biosciences) in order to remove any unbound antibody, and finally checked for viability and counted, as described before.

**Table 1:** AbSeq antibodies used for multi-omics analysis

Abseq	Cat #	Clone
CD3	940,000	SK7
CD8	940,003	RPA-T8
CD14	940,005	MΦP9
CD56	940,007	NCAM16.2
CD143	Custom	BB9
CD19	940,247	HIB19
CD4	940,001	SK3
CD45	Custom	2D1
HLA-DR	940,010	G46-6
CD11c	940,024	B-ly6
CD36	940,224	CLB-IVC7
CCR2	940,286	LS132.1D9
CD64	940,262	MD22
Siglec-7	Custom	F023-420
CD57	Custom	QA17A04
NKG2C	Custom	134,591
NKG2A	Custom	131,411

#### Single cell capture and cDNA synthesis

These steps were carried out by following the Manufacturer’s protocol for Single Cell Capture and cDNA Synthesis with the BD Rhapsody™ Single-Cell Analysis System. Briefly, cells were resuspended in 650 µl of cold BD Sample Buffer at a concentration ranging from 20 to 30 cells/µl, for an estimated capture rate of 10,000–15,000 single-cells, and then immediately loaded on a BD Rhapsody cartridge (BD Biosciences, cat# 633733) by using the cartridge reagent kit (BD biosciences, cat# 633731) for single-cell capture with the BD Rhapsody™ Scanner. After that, loading and washing Cell Capture Beads were performed before proceeding with cell lysis and beads retrieval. Finally, mRNA reverse transcription into cDNA was carried out by using the BD Rhapsody cDNA Kit (BD Biosciences, cat# 633773).

#### cDNA libraries preparation and next generation sequencing (NGS)

cDNA libraries preparation was performed by using the BD Rhapsody Targeted mRNA and AbSeq Amplification Kit (BD Biosciences, cat# 633774), according to the Manufacturer’s instructions. cDNA was amplified for 12 cycles using the pre-designed BD Rhapsody Immune Response Panel Hs (BD Biosciences, cat# 633750) containing primer pairs that target 399 genes commonly expressed in human immune cells. The resulting mRNA Targeted, Sample Tag and AbSeq libraries were quantified by Qubit 4 Fluorometer (ThermoFisher Scientific) before proceeding with sequencing procedures.

The cDNA libraries first underwent quality control and were subsequently deep-sequenced in 100 bp paired-end mode on a NovaSeq 6000 Sequencing System (Illumina) at CIBIO’s Next Generation Sequencing Core facility (University of Trento, Italy).

### Bioinformatic analysis

#### Pre-processing

Raw fastq files were processed using the Rhapsody analysis pipeline on the Seven Bridges Genomic platform (BD Biosciences). Paired-end reads were filtered based on quality (score < 20) and read length (R2 < 64 bp). Valid reads were mapped to the targeted gene panel reference and AbSeq reference using Bowtie2 (Version 2.5.1) and underwent universal recursive substitution error correction (RSEC) and distribution-based error correction (DBEC - for genes with RSEC-corrected depth > 4). Sample tag assignment involved quantifying reads for each tag on every cell, with cells assigned to a sample if the minimum read count for one tag was met. Multiplets were identified if counts for more than one tag were reached, and cells not meeting the threshold were labeled undetermined. Both multiplets and undetermined cells were excluded from downstream analysis.

#### Normalizing and thresholding

The resulting unique molecular identifier (UMI) count table was imported into R (v.4.2.0) and processed with Seurat (v.4.1.1) [13]. Each sample was assessed individually before integration. Cells with reads of genes less than 2,000 were retained for further analysis. RNA and AbSeq were normalized separately. For gene expression, a natural log normalization using a scale factor of 10,000 was performed across the library for each cell. For AbSeq, CLR centered log ratio (CLR, margin = 2) was applied for each cell.

#### Cell type annotation

The cells were annotated using the function `FindTransferAnchors` to remove any potential contaminants with default parameters. The reference used was the pbmc3k data set, a single-cell RNA-seq data set on peripheral blood mononuclear cells (PBMC) generated by 10X Genomics and publicly available on their website (https://support.10xgenomics.com/single-cell-gene-expression/datasets). Only cells annotated as NK were retained for downstream analysis. Normalized UMI counts were scaled and input to calculate principal components (PCA). The first 15 PCAs were used for clustering with a resolution of 0.5.

#### Clustering and downstream data analysis

In order to identify subpopulations of cells and differential gene expression in the NK population, the scRNA data were analysed with Seurat (version 4.2). Prior to clustering the data, we ensured that the data were batch-corrected using the “IntegrateData” function of the Seurat package.

Cell clustering was performed using the “FindNeighbors” and “FindClusters” functions using the original Louvain algorithm. The resulting clusters were visualized using the Uniform Manifold Approximation and Projection algorithm (UMAP) and the DimPlot function with reduction = “umap”. For each cluster, markers were discovered using the “FindAllMarkers” function, which identifies genes that are differentially expressed between each cluster and all other cells. Markers were identified based on a threshold of adjusted p-value < 0.05 and log2 fold change > 0.25.

To determine differential expression (DE) among the three groups of patients (*Naïve*, *Healed* and *Vaccine groups*), differential expression analysis was performed using the “FindMarkers” function with Wilcoxon rank sum test for each pairwise comparison with default parameters. Significant genes were selected based on an adjusted p-value (Bonferroni corrected) threshold of < 0.25 and |log2 fold change| > 0.25.

Pathway enrichment was investigated by Gene Ontology (GO) over-representation analysis using the enrichGO function implemented in the clusterProfiler R package (version 4.10.1).

## Results

### Integration of NK transcriptomes from 9 individuals revealed 5 distinct NK clusters

We initially profiled fresh human peripheral blood samples collected from the 9 enrolled subjects (mean age: 48.1 ± 9.7 years; age range: 27–62 years) belonging to the *Naïve group* (individuals who were never infected by SARS-CoV-2), to the *Healed group* (patients who were affected by COVID-19 and already recovered) and to the *Vaccine group* (subjects who received vaccination against the virus). A summary of patients’ characteristics is outlined in Table 2.

**Table 2:** Baseline demographic characteristics for the study participants

Patient #	Sex	Age	Antibody titer or AU/ml	COVID-19 symptoms
*Naïve Group*				
1	M	48	-	-
2	M	62	-	-
3	M	45	-	-
*Healed Group*				
1	M	55	1:320	flu-like
2	M	49	1:160	flu-like
3	M	27	-	flu-like
*Vaccine Group*				
1	M	54	1:320	-
2	F	44	1000AU/ml	-
3	M	49	-	-

**Fig. 1:**
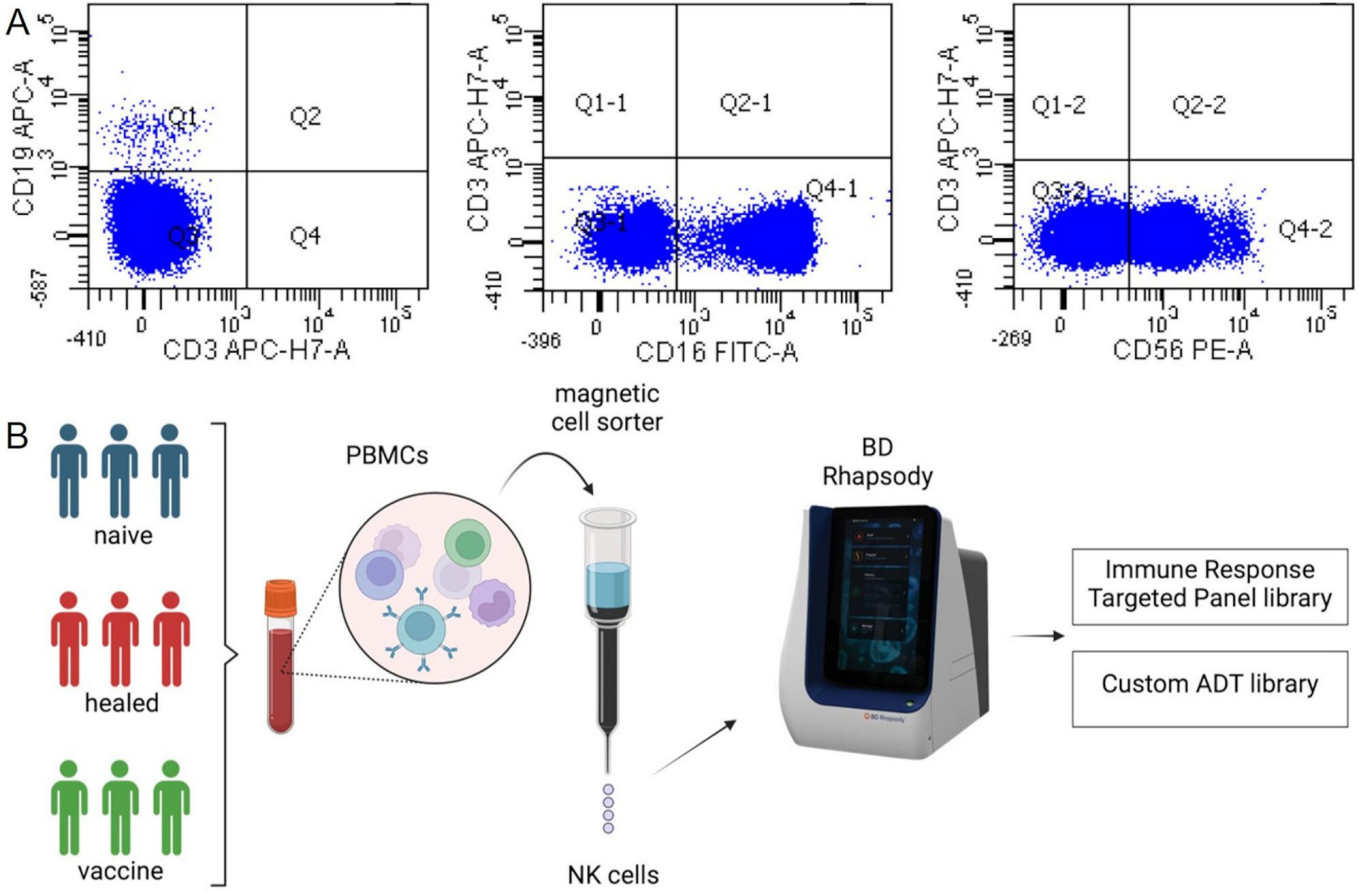
NK cell isolation and study design. (**A**) Verification of correct NK cell enrichment in patients’ blood samples. The plots are representative of all patients and demonstrate the enrichment of cells populations with phenotype CD3^−^/CD16^+^/CD56^+^. (**B**) Schematic overview of the study design (Created with BioRender.com)

NK cells were enriched and analysed for their specific immunophenotype by flow cytometry. This analysis demonstrated the successful enrichment of cell populations with phenotype CD3^−^/CD16^+^/CD56^+^ by all blood samples (Fig. 1A). The gating strategy was reported into the Additional file 1 for completeness. Enriched NK cells were then subjected to targeted scRNA-seq using the BD Rhapsody platform (Fig. 1B).

After rigorous quality control (QC) processing, a total of 6,005 high-quality single transcriptomes were retained. Of these, 2,029 cells were from the *Naïve group* (*N* = 3), 1,797 cells were from the *Healed group* (*N* = 3), and 2,179 cells were from the *Vaccine group* (*N* = 3). The results of the QC process are reported in Additional file 1.

**Fig. 2:**
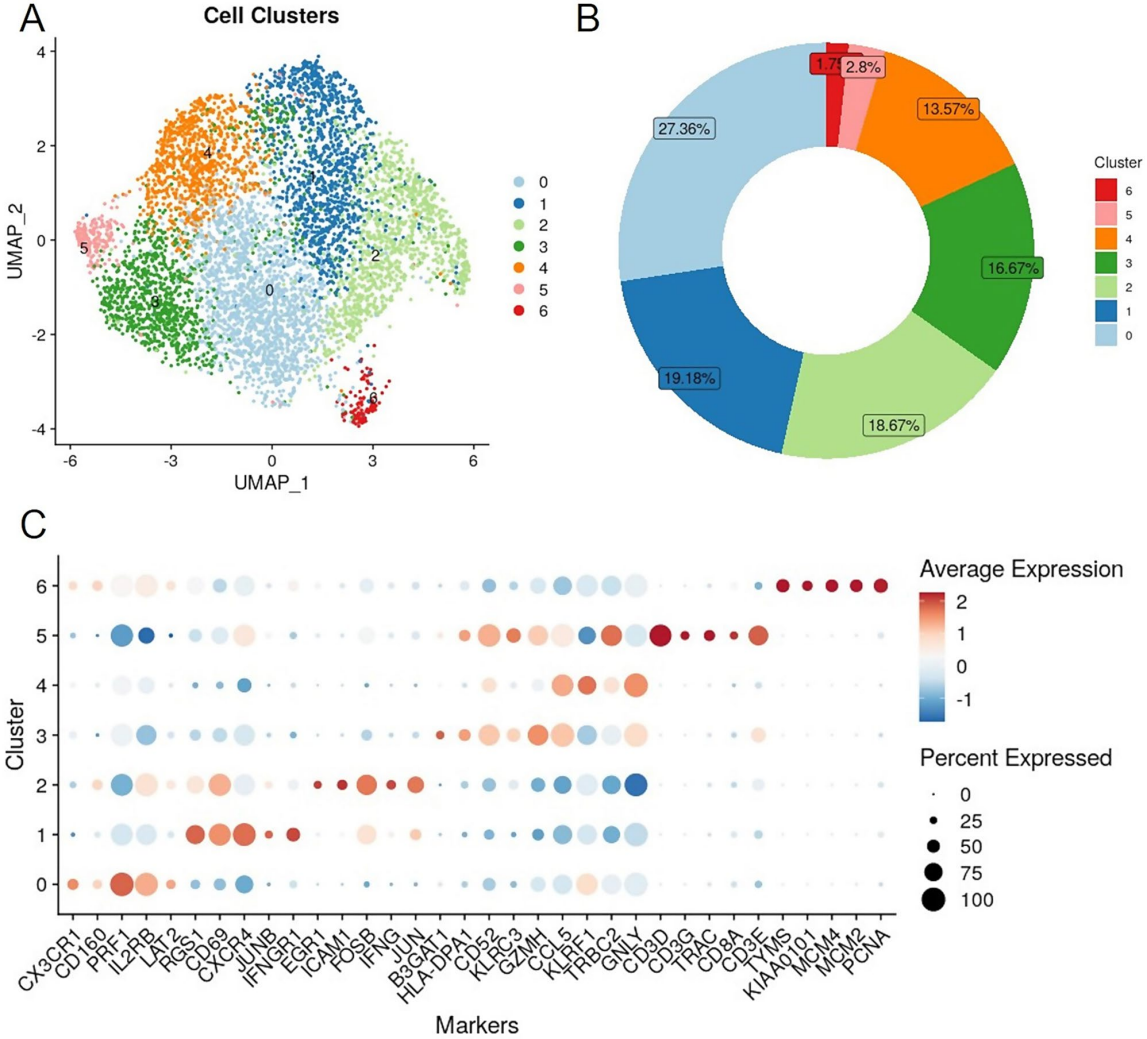
NK cell sub-populations. (**A**) UMAP projection of all retained NK cells. (**B**) Proportion of cells in each cluster. (**C**) Top 5 up-regulated markers per cluster

Integration of the single-cell transcriptomes from different individuals revealed seven unique NK cell sub-populations, which were visualized using uniform manifold approximation and projection (UMAP) (Fig. 2A). Differential expression analysis identified conserved markers for each cluster, regardless of group allocation (Fig. 2C). Of note, clusters 5 and 6 together represented less than 5% of the total cells (Fig. 2B). Furthermore, these clusters exhibited distinct transcriptional profiles compared to the other clusters. Cluster 5, accounting for 2.8% of the total, expressed *CD3D*,* CD3G*, and *TRAC*, suggesting the presence of T cells or NKT cells. Additionally, Cluster 6, comprising 1.8% of the total cell population, showed elevated expression of DNA repair and apoptosis genes such as *TYMS*,* KIAA0101*, and *MCM4*, possibly indicating stress induced by the isolation protocol. Consequently, Clusters 5 and 6 were excluded from further analysis, resulting in five NK cell clusters being retained for investigation.

### NK clusters characterization revealed presence of Active and Mature NK cells

**Fig. 3:**
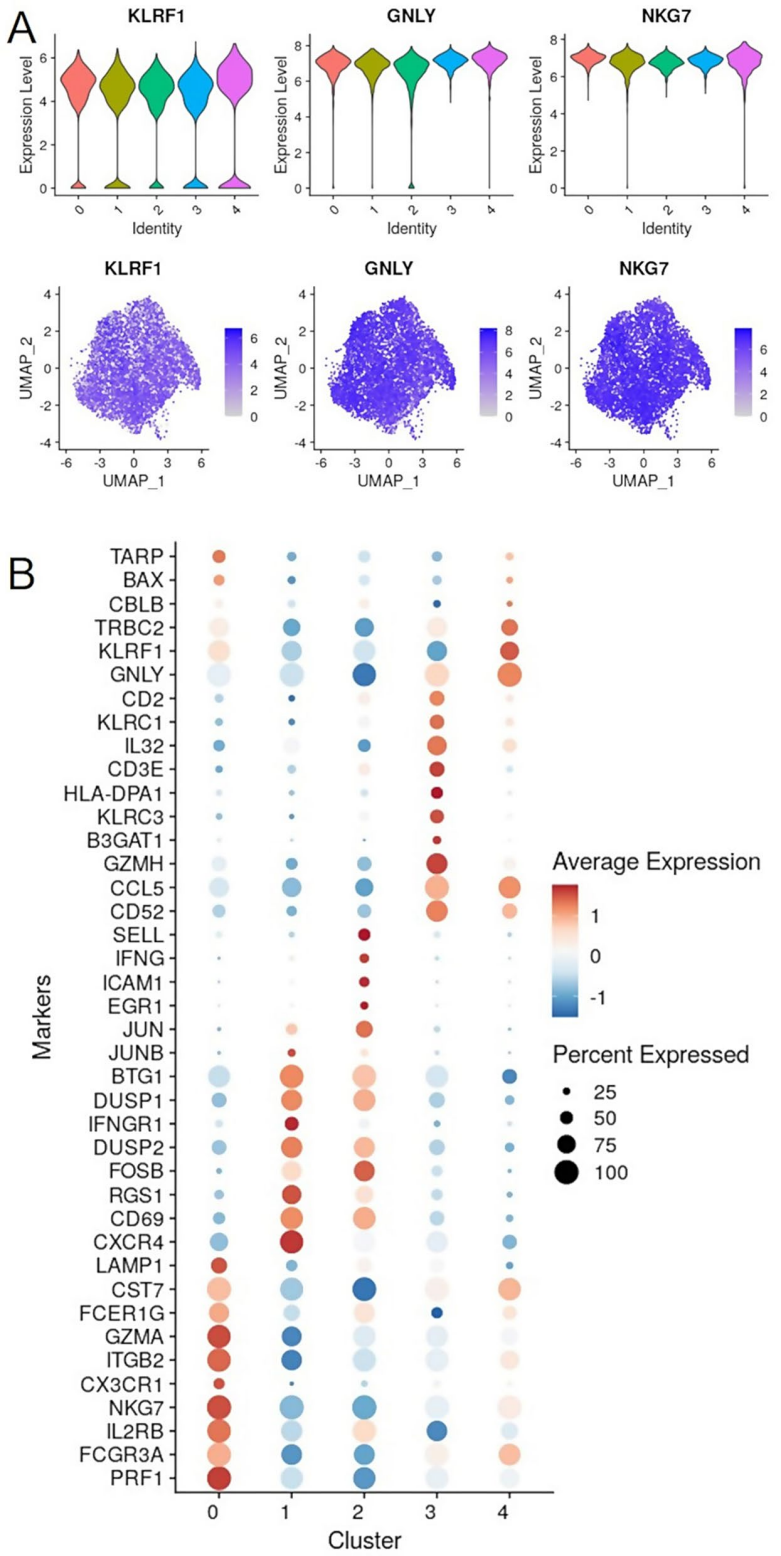
Characterization of NK clusters. (**A**) Expression of NK cell markers per cluster. (**B**) Top 10 mRNA markers per cluster

We next set out to characterize the 5 distinct NK clusters. Consistently, all remaining clusters exhibited high expression of NK cell lineage-defining markers *KLRF1*,* NKG7*, and *GNLY* (Fig. 3A). For each cluster, the top five up-regulated differentially expressed genes (DEGs), ranked by fold change, were visualized using a heatmap (Fig. 3B). The heatmap reveals that clusters 1 and 4 share a similar fundamental pattern, while the other clusters are transcriptionally distinct.

**Fig. 4:**
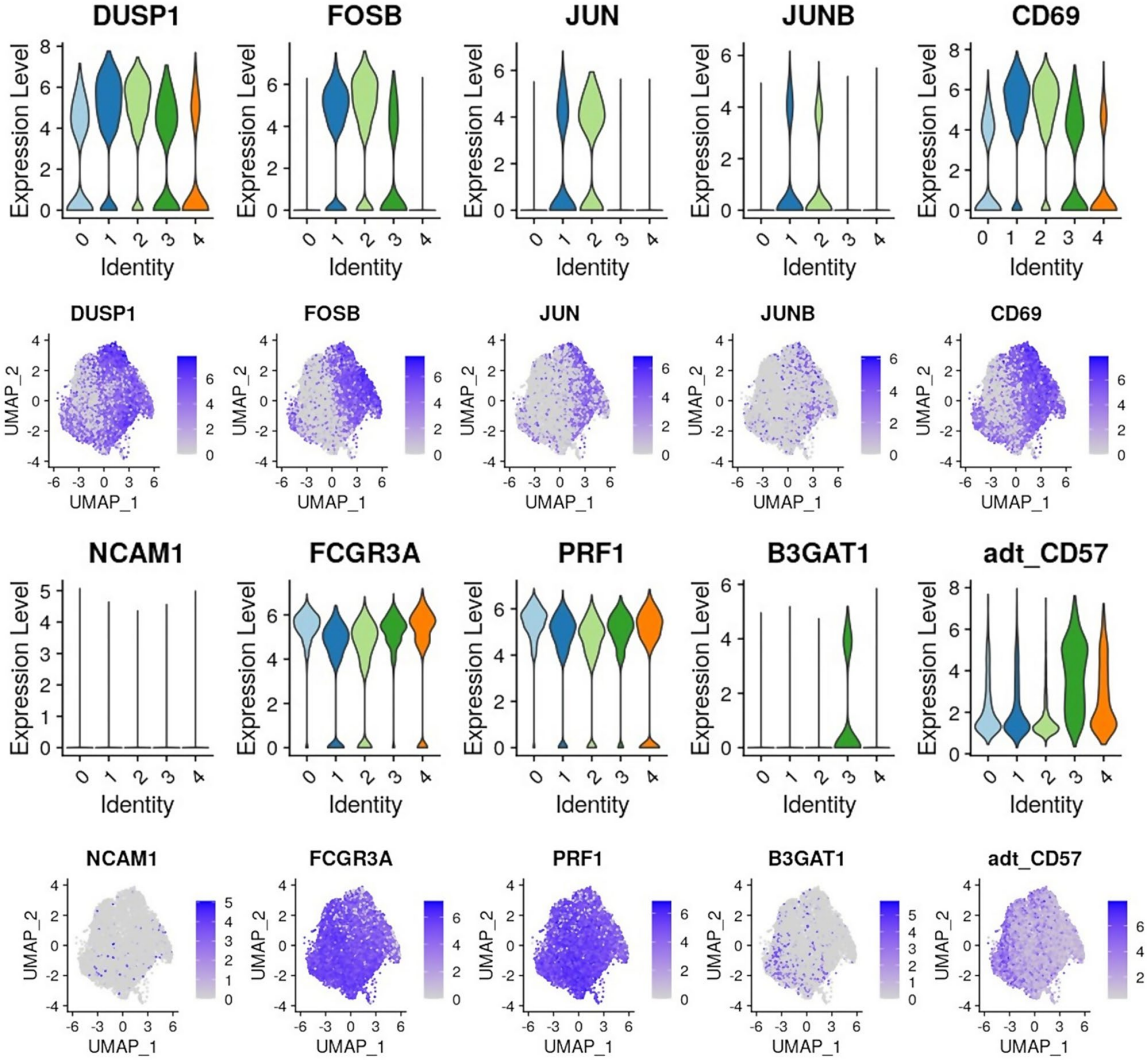
Key NK cell markers. Expression of NK subpopulation cell makers per cluster

Interestingly, Yang et al. [14] identified five clusters of human NK cells by scRNA seq analysis of blood samples. Based on specific marker expression, these NK clusters were designated as “CD56bright NK”, “Transitional NK”, “Active NK”, “Mature NK”, and “Terminal NK”. In our study, NK clusters 1 and 2 exhibited higher expression levels of several immediate early genes (IEGs), including *FOSB*,* JUN*,* JUNB*,* CD69*, and DUSP1, suggesting that these clusters might represent Active NK cells (Fig. 4).

*B3GAT1* (CD57), a marker typically associated with terminally mature NK cells, was predominantly expressed in cluster 3, both at the mRNA and protein levels. Cluster 4 also showed intermediate expression of CD57 at the surface level, as well as *PRF1* and *FCGR3A* (CD16), suggesting that this cluster might represent Mature NK cells. Cluster 0 shared the expression of key markers with clusters 1 and 2 (e.g., *CD69*,* DUSP1*) and clusters 3 and 4 (e.g., *PRF1*,* FCGR3A*), displaying intermediate characteristics between the active and mature genotypes. Finally, as reported in Fig. 4, the abundance of NCAM1 (CD56) transcripts was very low, making it unsuitable for identifying CD56 ^bright^ NK cells.

### DEG analysis revealed a more activated NK phenotype in vaccinated patients

**Fig. 5:**
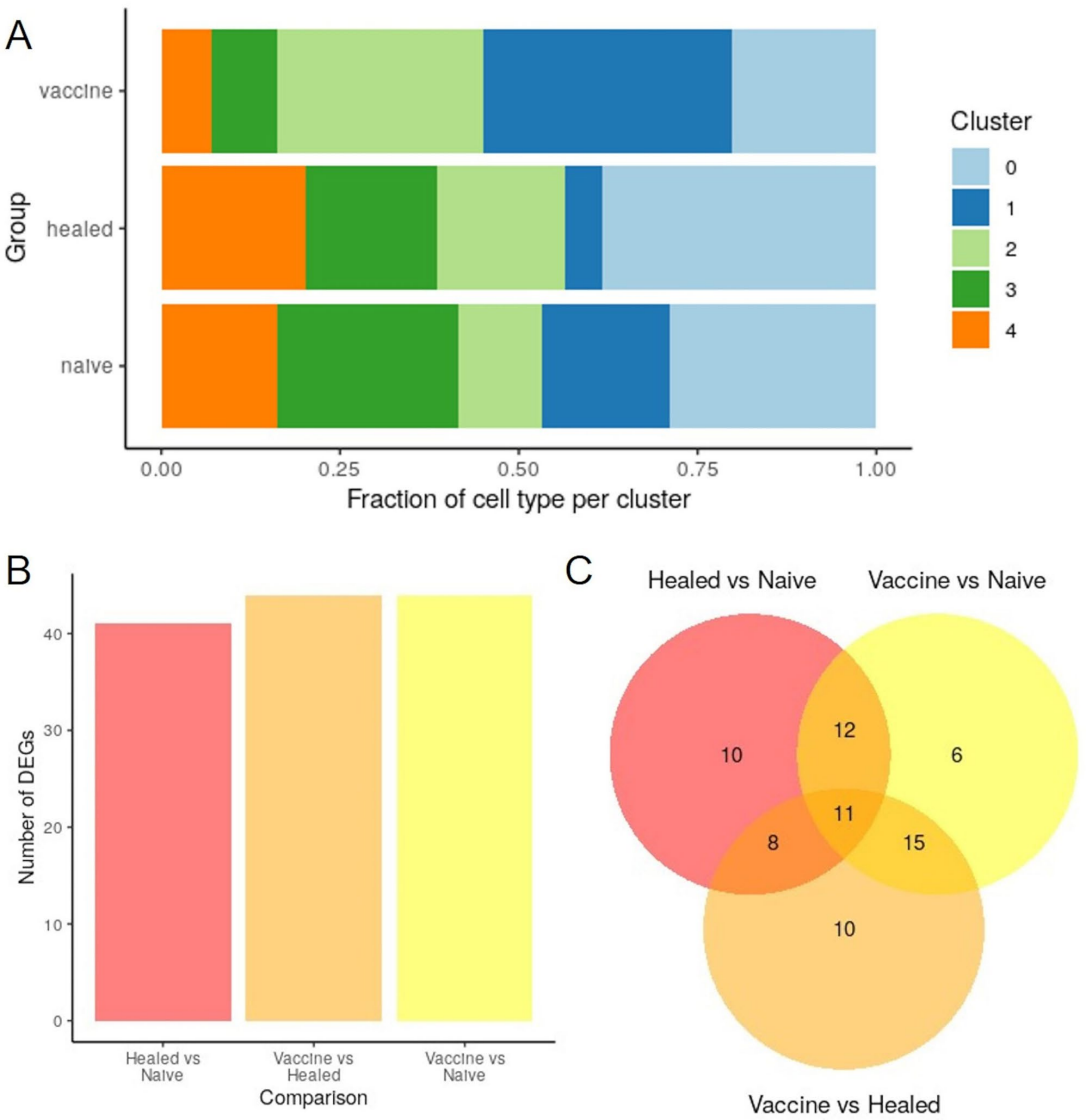
NK cluster and study groups. (**A**) Fraction of cell type in each cluster for the *Naïve*, *Healed* and *Vaccine groups*. (**B**, **C**) Overview of the differentially expressed genes from pairwise comparisons between *Naïve*, *Healed* and *Vaccine groups*

By calculating the relative proportion of each cluster within patient groups, we verified that cells from all three groups contributed to each cluster. However, notable differences were observed among the groups. Specifically, more than 40% of the *Naïve group* cell population belonged to clusters 3 and 4 (i.e., Mature NK cells), while over 75% of the Vaccine group cell population was found in clusters 0, 1, and 2 (i.e., Active NK cells). The *Healed group* exhibited an intermediate phenotype between Active and Mature NK cells (Fig. 5A).

To investigate changes in gene expression profiles within each patient group, pairwise differential expression analysis was conducted. Differentially expressed genes (DEGs) were identified by pooling cells from samples of the same group, resulting in three comparisons: *Healed group vs. Naïve group*,* Vaccine group vs. Naïve group*, and *Vaccine group vs. Healed group*. Using a q-value threshold of 0.25 and a log2FoldChange threshold of |0.25|, we identified 41 DEGs for the *Healed group vs. Naïve group*, 44 DEGs for the *Vaccine group vs. Naïve group*, and 44 DEGs for the *Vaccine group vs. Healed group* (Fig. 5B).

Many DEGs were shared between the *Vaccine group vs. Naïve group* and *Healed group vs. Naïve group* comparisons, as well as between the *Vaccine group vs. Healed group* and *Vaccine group vs. Naïve group* comparisons (Fig. 5C).

**Fig. 6:**
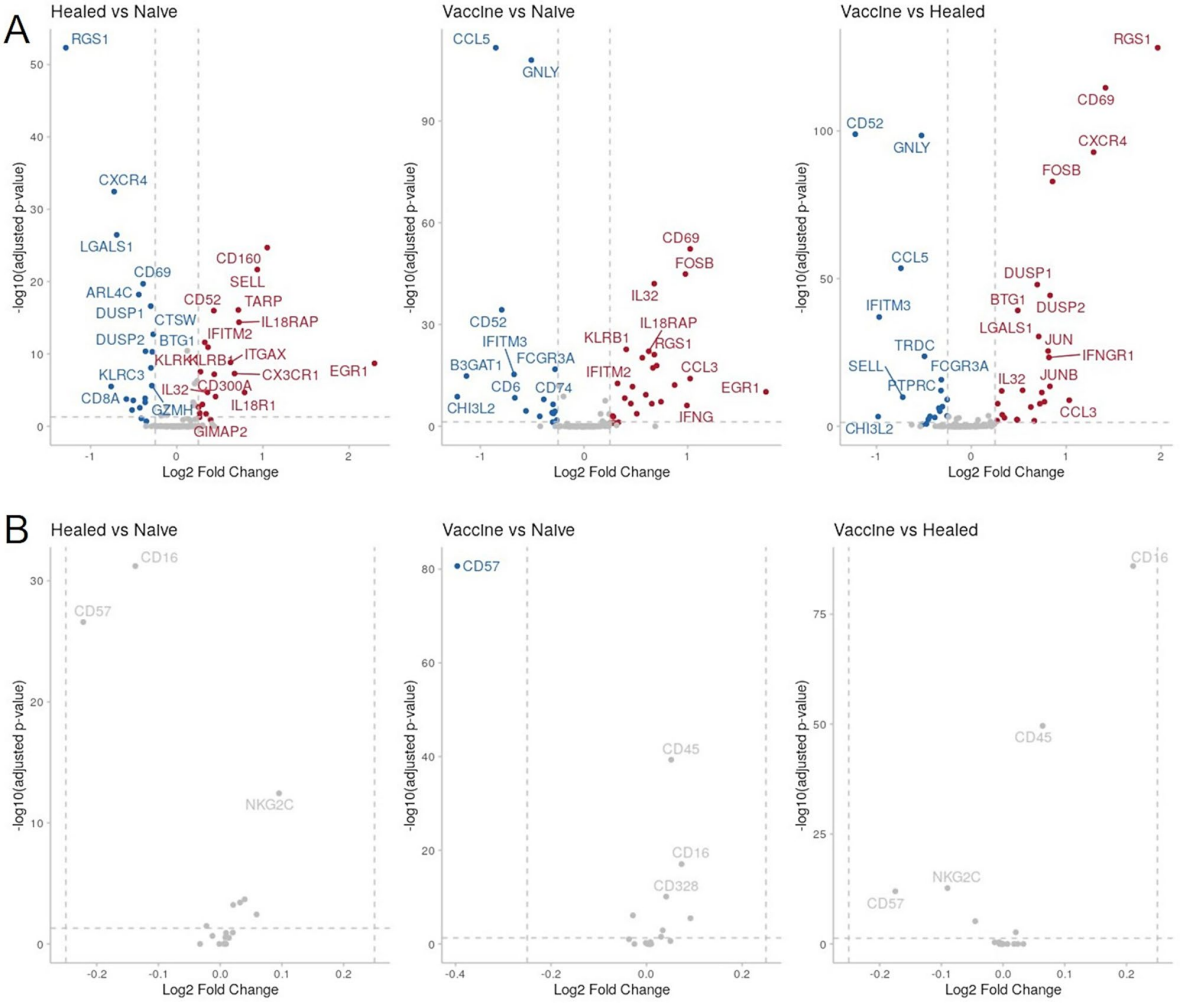
Differentially expressed markers among study groups. (**A**) Volcano plots of the differentially expressed genes from pairwise comparisons between *Naïve*, *Healed* and *Vaccine groups*. (**B**) Volcano plots of the differentially expressed surface proteins (ADT) from pairwise comparisons between *Naïve*, *Healed* and *Vaccine groups*

DEGs resulting from the aforementioned pairwise comparisons are presented in Fig. 6A. The same comparisons were utilized to identify differentially expressed surface proteins using antibody-derived tag (ADT) data, as shown in Fig. 6B.

### Antibody levels influence gene expression of NK cells

#### Clustering of pseudobulk expression

**Fig. 7:**
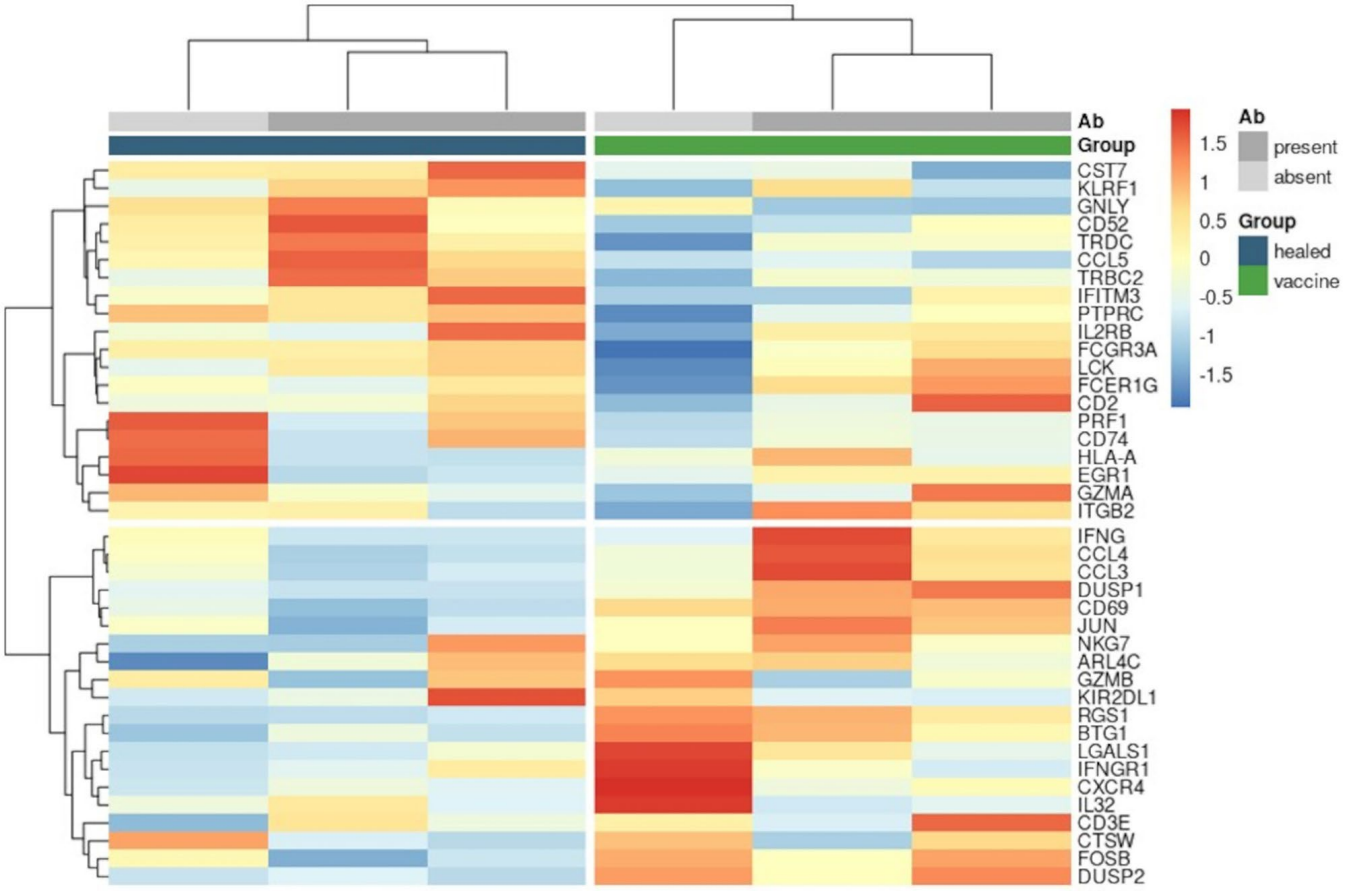
Antibody development and NK cell gene expression. Unsupervised clustering (distance: euclidean - method: ward D2) of pseudobulk expression of the top 10% most variable genes

**Fig. 8:**
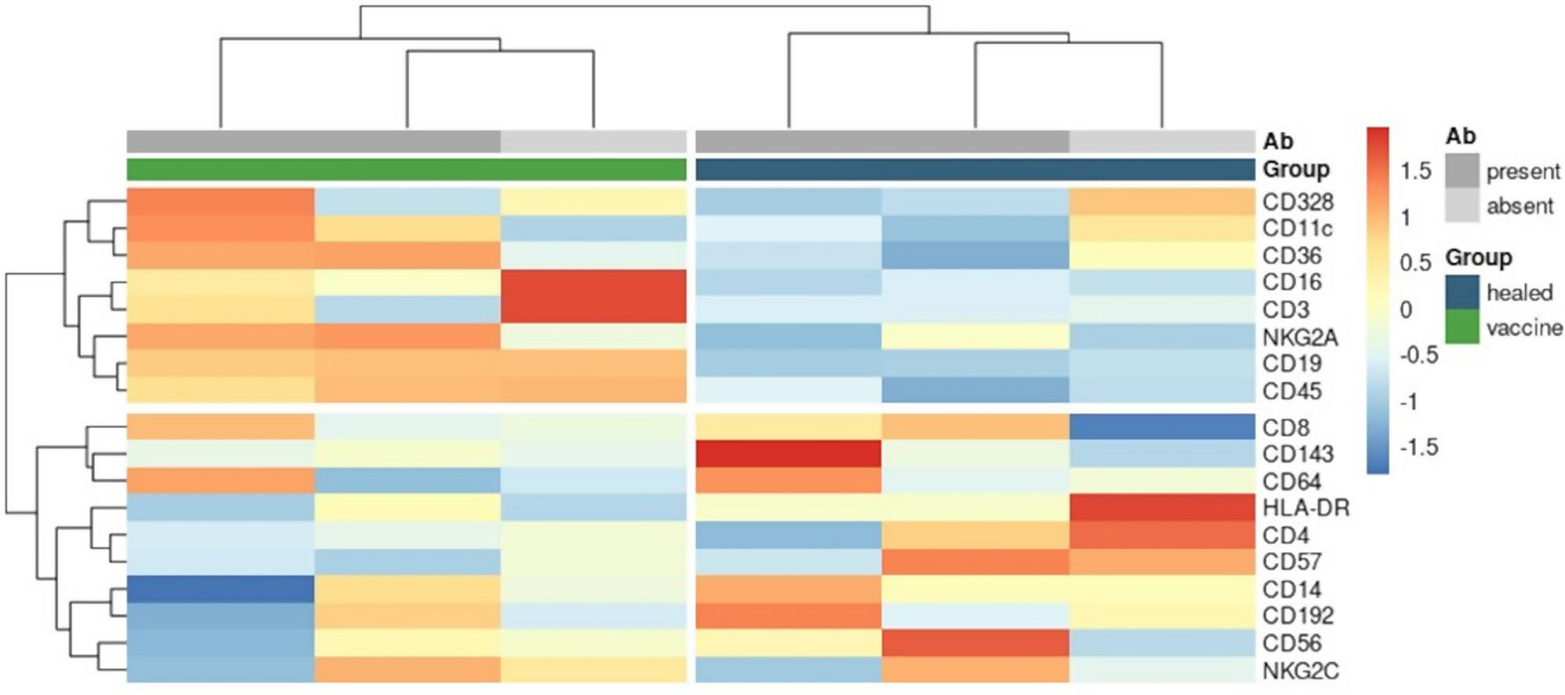
Antibody development and NK cell protein expression. Unsupervised clustering of pseudobulk expression of all ADTs

To investigate the potential correlation between gene expression and antibody levels in healed and vaccinated patients, each group was stratified into two subsets based on protective antibody levels. Patients with antibody levels ≥ 1:160 were categorized as “responders” (R), while those with lower levels were categorized as “non-responders” (NR). Unsupervised clustering of pseudobulk expression of the top 10% most variable transcripts revealed that healed and vaccinated patients tended to cluster together with others from the same group, regardless of antibody levels (Fig. 7). Similar clustering patterns were observed when analysing average expression levels of all detected antibody-derived tags (ADTs) (Fig. 8).

#### Differential expression and pathways enrichment

**Fig. 9:**
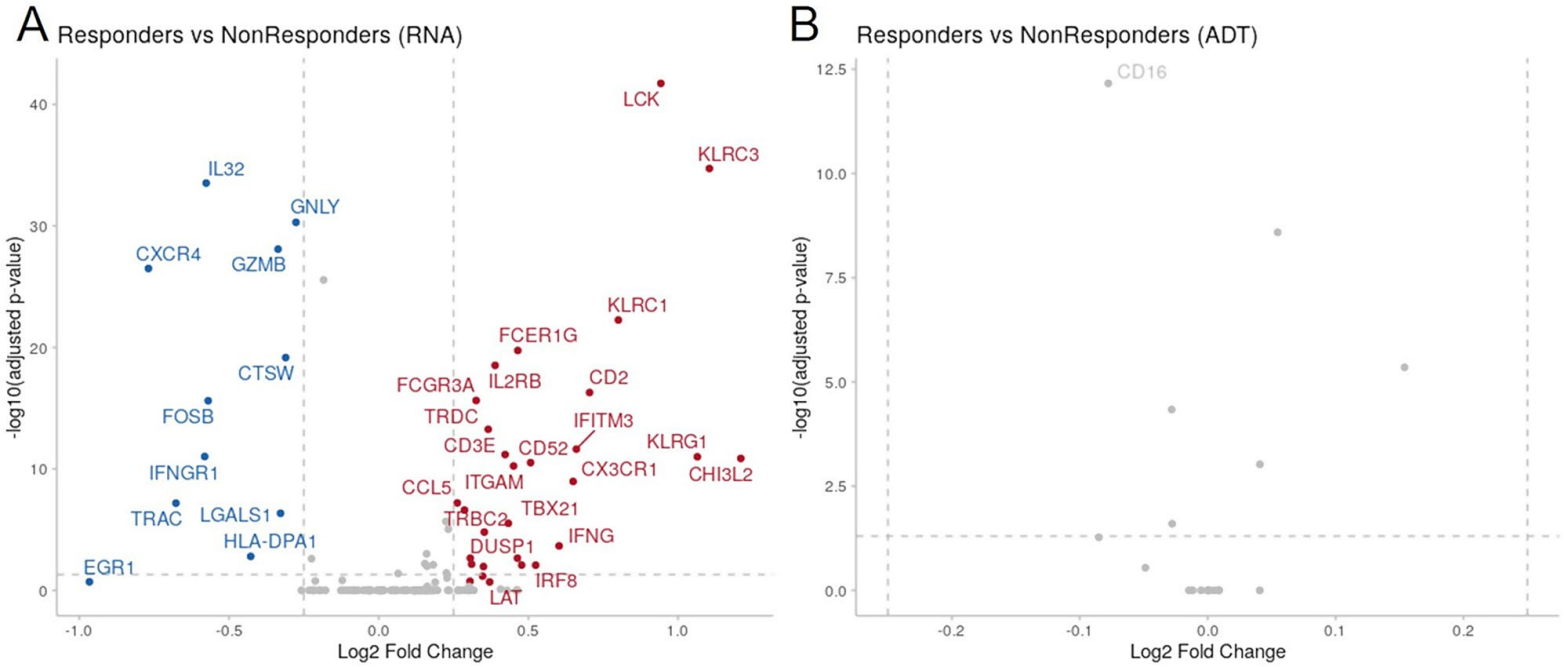
Differential gene expression in responders and non-responders subjects. Overview of the differentially expressed genes from pairwise comparisons between responders and non-responders (transcripts on **A**, ADTs on **B**)

To investigate significant changes in NK gene expression profiles between responders and non-responders, we conducted pairwise differential expression analysis. Differentially expressed genes (DEGs) were identified by aggregating cells from samples within each group. Using an adjusted p-value threshold of 0.25 and a log2FoldChange threshold of |0.25|, we identified 41 differentially regulated transcripts, while no differentially regulated antibody-derived tags (ADTs) were found, as depicted in the volcano plots (Fig. 9).

The 41 identified DEGs were subjected to Gene Ontology (GO) over-representation analysis, which revealed significant enrichment of four GO terms under the Molecular Function (MF) ontology, with adjusted p-values ≤ 0.25. These enriched pathways are detailed in Table 3. A comprehensive list of genes associated with each pathway can be found in the Additional file 2.

**Table 3:** List of significantly enriched GO terms

ID	Ontology	Description	*p* value	*P* adjust
GO:0019901	MF	protein kinase binding	0.0009	0.0937
GO:0016004	MF	phospholipase activator activity	0.0015	0.0937
GO:0060229	MF	lipase activator activity	0.0015	0.0937
GO:0019900	MF	kinase binding	0.0018	0.0937

## Discussion

Current research in COVID-19 patients contributed to reveal a dysregulated immune system with hyper-inflammatory responses and lymphopenia [15–17]. Nevertheless, changes in cell populations responsible for the innate immune response to the virus deserve to be furtherly clarified.

Innate immunity assures for the first defense mechanism against virus infections, by recognizing pathogen organisms/molecules and triggering signaling pathways mediated by the release of interferons, cytokines and chemokines. These signaling factors control the activation and recruitment of defense cells like neutrophils, monocytes/macrophages, and Natural Killer (NK) cells, with the role of eliminating pathogens [18].

Exerting a crucial role in the innate defense against viral infections and malignancies, NK cells are likely involved also in the first responses to COVID-19 disease. SARS-CoV-2 infection causes the secretion of proinflammatory cytokines and the recruitment of proinflammatory cells, which activate a systemic inflammatory reaction called macrophage activation syndrome (MAS) or - more commonly - “cytokine storm” (CS) [19, 20]. This may result in the regulation of specific molecular targets for NK cell activation to modulate viral load in the patient and avoid a hyper-inflammatory syndrome. Despite several studies focusing on COVID-19 effects on the innate immune system, the NK cell behaviour in SARS-CoV-2 infection deserves to be better investigated. Collecting more data on transcriptome and immunophenotype changes into NK cells of COVID-19 patients may be crucial to improve the clinical approaches for the prevention of virus infection and treatment of COVID-19 disease.

In this scenario, the recent advancements in multi-omics technologies providing detailed cell-level analysis, allowed to shed some light on the immune dysregulations associated with COVID-19, contributing to a deeper understanding of the immune system’s role in the development, progression, and resolution of this pathology [21, 22] (reviewed for example by Müller et al. [23] and Montaldo et al. [24]). However, current literature on the topic is still lacking single-cell multi-omics analyses specifically focused on NK cells as key actors of the innate immune response towards SARS-CoV-2 infection. In this study, the BD Rhapsody™ technology was used to conduct the multidimensional transcriptomics (whole transcriptomic analysis, WTA), and surface marker analysis (antibody sequencing, AbSeq) of NK cell populations isolated from COVID-19 infected and recovered individuals, in comparison with patients who received the vaccine against the virus and healthy donors who have never been infected or vaccinated. To the best of our knowledge, only one study can be found in the literature which used the BD Rhapsody™ platform for the single-cell multi-omics investigation of the whole fraction of peripheral blood mononuclear cells (PBMCs) isolated from COVID-19 patients. This work by Soni and colleagues [25] detected high expression of cell identity and regulatory markers (i.e., CD3E, CD4, CD8A, CD5, CD7, GITR, and KLRB1) in healthy individuals, whereas markers related to cell activation (i.e., CD38, CD28, CD69, CD62L, CD14, and CD16) were upregulated in the SARS-CoV-2 infected patients at both mRNA and protein levels. Additionally, an over-expression of cytokine and chemokine receptors (i.e., CCR5, CCR7, CCR4, CXCR3, and PTGRD2) was observed in recovered individuals. Besides defining mRNA and protein profiles of the PBMC fraction in COVID-19 patients *versus* healthy subjects, this study also underlines that oligo-based methods which identify cell surface markers related to specific pathological states can help in disease diagnosis, prognosis, and therapy [25].

Focusing specifically on Natural Killer lymphocytes enriched from the peripheral blood of enrolled subjects, the present research identified 5 specific clusters, which can mainly be ascribed to 2 subsets of NK cells, namely Active NK cells (clusters 1 and 2) and Mature NK cells (clusters 3 and 4). Moreover, analysing the cluster distribution among the 3 experimental groups, the *Vaccine group* cell population contributed mainly to the Active NK clusters, whereas the *Naïve group* cells mainly belonged to the Mature NK clusters. Regarding the fifth cluster, cluster 0, it seems to exhibit intermediate characteristics between the active and mature genotypes.

Active NK cells were previously defined by Yang and colleagues [14] as transcriptionally active cells showing the activation of key signaling pathways, such as (i) the KRAS-MAPK pathway, which regulates cell proliferation, differentiation and survival [26], and (ii) the TRAF6-NF-κB pathway, which triggers antiviral innate immune responses through the induction of proinflammatory cytokine release [27]. The existence of this cluster suggests homeostatic activation of NK cells, which may influence their survival, proliferation, or differentiation [14]. Considering that, the detection of NK cells in this active state into the blood of vaccinated patients is particularly significant, since it may be due to the stimuli regarding COVID-19 vaccination acceptance.

On the contrary, blood samples from healthy donors (*Naïve group*) who were never affected by COVID-19 and were not vaccinated, were mainly characterized by the presence of Mature NK cells, which are the cell subset in the final stages of development, with the acquisition of functional competence in cytotoxicity and production of interferon (IFN)-γ [14, 28, 29]. Given the lack of expression of CD69 as a marker of recent stimulation, Mature NK cells forming clusters 3 and 4 can be considered in a resting state [30], which is consistent with their prevalence in the blood of non-stimulated healthy individuals.

Interestingly, clustering of NK cells from patients belonging to the *Healed group* showed intermediate characteristics between the Active and the Mature subsets, which may be consistent with a condition of infected and then recovered subjects. Indeed, this cluster profile suggests both an activation state of NKs due to the stimulation by the virus and a resting state of NKs related to the recovered condition from the disease.

The analysis of DEGs within the 3 experimental groups underlined NK cells different responses based on the patients’ condition. Compared to the healthy donors, subjects from the *Healed group* showed the upregulation of markers like CD160, CD52, EGR1 and SELL. Interestingly, CD160 has been recognized as an activating NK cell receptor capable of increasing cytokine production in response to specific ligation of viral antigens [31]. Its upregulation in convalescent COVID-19 subjects has been previously demonstrated, detecting a significant increase in moderate rather than severe illness [32]. This suggests a role for CD160 in controlling disease state through direct cytotoxicity [33]. The upregulation of CD52 may also have implications in the innate immune response to SARS-CoV-2 infection. This NK cell ligand is able to bind to Sialic-acid-binding immunoglobulin-like lectins (Siglecs), driving the inhibition of damage-associated molecular pattern (DAMP)-mediated inflammation and so potentially exerting a protective effect towards COVID-19 disease [34]. The transcriptional regulator EGR1 is one of the Early growth response (EGR) family genes belonging to the group of Immediate-early genes (IEGs), which are stress-related genes responsible for the reaction to various external stimuli, including virus infections [35]. EGR1 is known to regulate inflammation and immune response, driving the production of proinflammatory cytokines (i.e., interleukins and tumor necrosis factor) and chemokines. This transcription factor was demonstrated to be significantly upregulated in cells infected with different coronavirus strains, such as the gammacoronavirus IBV (infectious bronchitis virus), the alphacoronavirus HCoV-229E (human coronavirus-229E), and the betacoronavirus HCoV-OC43 [35]. Of note, EGR1 was indicated as one of the most highly expressed transcription factors also in COVID-19 patients [36], representing one of the markers related to the first immune response to the infection.

Finally, SELL (CD62L) gene was found to be expressed by mature NK cells with polyfunctionality, which combine the capacity of CD56^bright^ NK subsets to produce INF-γ in response to cytokines and to proliferate in vivo during viral infection, with the cytotoxic ability - typical of CD56^dim^ populations - to kill and produce cytokines upon engagement of activating receptors [37]. Remarkably, Yang and colleagues [14] identified CD62L among the markers expressed at higher levels by Active NK cells.

Overall, these upregulated genes characterizing NK lymphocytes from COVID-19 recovered patients *versus* healthy donors turn out to be cell activation markers with an elevated expression that signifies their involvement during the early infection phase.

Considering the *Vaccine versus Naïve groups* comparison, the panel of DEGs with upregulated expression included, among others, CD69, FOSB, IL32, CCL3 and EGR1. The high expression of CD69, which we have already mentioned as the key marker of Active NKs, is consistent with the identification of NK cell clusters in the activate state within the blood of vaccinated subjects. Similarly, also FOSB is recognized as a marker of Active NK cells. Belonging to the very early activator protein (AP)-1 transcription factor family, its upregulation is correlated to natural cytotoxicity exerted by the human NK lymphocytes upon stimulation [38]. Notably, CD69 and FOSB are the most significantly upregulated genes in the *Vaccine group*.

The chemokine CCL3 is one among the several chemokines which are released by NK lymphocytes during immune response to infection. In post-COVID-19 patients, CCL3 was found to maintain higher expression than in healthy controls [39]. Regarding IL32 upregulation after vaccine administration, Zamani and co-workers [40] have recently demonstrated its predicting role in determining the severity and outcome of COVID-19 disease. Its increased expression in vaccinated subjects, together with CCL3 upregulation and EGR1 activation like in the *Healed group*, suggests that the vaccine might act regulating some key markers which are modulated by the virus as well.

Comparing the *Vaccine* and the *Healed groups*, significant upregulation of the markers CD69 and FOSB was found within the NK populations of vaccinated individuals, confirming the presence of a more activated cell subset into the blood of these patients *versus* the blood of recovered subjects. Consistently, the marker DUSP1, overexpressed by vaccinated patients, is among the IEGs which were reported to characterize the transcriptome of Active NK cells [14]. Additionally, the chemokine receptor CXCR4 resulted to be upregulated in NK cells from the *Vaccine group*. Together with CD69, FOSB and DUSP1, this gene was also reported to be upregulated in Active NK cells [14], but its expression is also known to decrease as the NK cell development proceed to a mature state [41]. This suggests that the Active NK cluster might not represent a unique developmental stage but might be a mix of different developmental stages receiving stimuli that produce their specific transcriptome profile [14].

Besides the transcriptome analysis to detect the DEGs among groups, some key membrane proteins were also demonstrated to be differently expressed according to the patients’ condition. Remarkably, subjects who recovered from virus infection showed a significative upregulation of NKG2C in comparison with healthy subjects. This membrane receptor was defined as a marker of Adaptive NK cells [14]. This unique subset of NK lymphocytes present characteristics of both the innate and adaptive immune system, exhibiting an increased capacity for IFN-γ production and antibody-dependent cellular cytotoxicity (ADCC) [42]. NKG2C can form dimers with CD94 and the resulting CD94/NKG2C heterodimer can bind to HLA-E. Hammer and colleagues [43] demonstrated that NK cells expressing the CD94/NKG2C receptor can specifically recognize the human cytomegalovirus peptides presented on HLA-E, leading to activation, expansion, and differentiation of adaptive NK cells. Regarding SARS-CoV-2, the literature reports contradictory results on the role of Adaptive NK cells in controlling virus infection and disease development [44]. On one hand, the activation of Adaptive NK cells was found to correlate with COVID-19 disease severity [8]. On the other hand, the absence of a gene encoding the NKG2C receptor in NK cells was shown to be a risk factor for severe disease development [45]. However, the presence of the NKG2C^+^ NK lymphocytes in recovered COVID-19 patients may suggest long-term impact of SARS-CoV-2 infection on NK cells, with a subpopulation that potentially suspend antiviral activity and acquire an adaptive profile. Indeed, previous evidence points to adaptive-like functionality of NK cells in response to viral infection, inflammation and cancer, suggesting that the inflammatory cytokines and viral products elicited by infections or vaccines can promote expansion, differentiation and persistence of Adaptive NK cells with enhanced effector function with respect to naive NK subsets [46].

Comparing the *Healed* and *Vaccine groups*, a subpopulation of NKG2C^+^ NK cells were also found into the blood of the vaccinated patients, but the marker was downregulated with respect to the recovered subjects. This may be ascribed to a stronger impact of the virus rather than the vaccine to induce memory-like NK cells with ADCC capacity.

The downregulation of CD57 NK cell marker was registered in the blood of both healed and vaccinated individuals in comparison with healthy donors. This membrane protein was established to identify terminally differentiated NK cells with increased cytotoxic activity [47], and its expression was shown to increase in case of chronic viral infections, particularly human cytomegalovirus infection [48]. On the other side, a recent clinical research by Savchenko and collaborators [49] reported a reduced level of CD57 expression in NK cells from patients with post-COVID syndrome before immune-rehabilitation through recombinant human IL-2 (rhIL-2), resulting to correct NK cell phenotype and functional activity. Based on these findings, the authors hypothesized a functional deficiency of NK cells in post-COVID syndrome. In this study, the decrease of CD57 expression in recovered and vaccinated individuals compared to the healthy subjects may be due to the higher representation of CD57-positive Mature NK cell clusters which were identified in the *Naïve group*. Additionally, since higher CD57 expression was found to correlate with a loss of reactivity to inflammatory cytokines [50], the lower positivity to this marker after exposure to viral antigens in recovered and vaccinated individuals may be consistent with the activation of NK cell subsets in response to the cytokine-mediated infection.

Interestingly, CD16 membrane protein resulted to be down regulated in the *Healed group* but upregulated in the *Vaccine group* in comparison with the *Naïve group*. The CD16 receptor mediates the NK cell activation, enabling them to detect and kill antibody-coated target cells via ADCC [39]. Considering that, its upregulation in vaccinated *versus* recovered patients is consistent with the more activated NK cell profile which was generally detected in the *Vaccine group*. Referring to previous literature on the topic, controversial data were reported about CD16 expression in COVID-19 disease. Recently, Soni and colleagues [25] highlighted an elevated CD16 expression among markers related to cell activation in SARS-CoV-2 infected patients. Conversely, other studies previously demonstrated that CD16 was downregulated in COVID-19 patients during the acute phase, as well as in convalescent subjects [51], suggesting a proinflammatory phenotype of NK cells during SARS-CoV-2 infection. Our data seem to be in line with these studies, detecting a decrease of CD16 expression in recovered individuals, but an increased expression likely due to the activation exerted by the vaccine administration.

Antibodies (Abs) are recognized as important immune mediators and potential diagnostic markers in several viral infections. Also in COVID-19 disease, humoral immunity or the development of effective antibodies against SARS-CoV-2 seem to exert a role in limiting disease burden in the community and in supporting the finding of new diagnostic, therapeutic, and vaccination solutions [52, 53].

SARS-CoV-2 is known to enter into host cells by the interaction of the receptor-binding domain (RBD) of the viral Spike (S) glycoprotein with the angiotensin converting enzyme-2 (ACE2), which functions as virus cell receptor on the surface of target cells [54]. Besides the S protein which is crucial for virus attachment and entry into the host cells, nucleocapsid (N), membrane (M), and envelope (E) are the other key structural proteins that can be targeted by the antibody response. The N protein was shown to be necessary for viral RNA synthesis, whereas the E and M proteins participate in viral assembly [53]. Antibody response to SARS-CoV-2 infection first develops against the N protein. However, protective immunity against SARS-CoV-2 infection was proved to mostly depend on the neutralizing antibody responses that target the S protein [55]. Most of neutralizing Abs act by recognizing the receptor-binding domain (RBD) of virus S protein to interact with viral particles and interrupt their binding to ACE2 receptor of infected host cells, thus mediating a reduction of viral infectivity [56]. Based on that, clinical trials testing therapies with convalescent plasma from recovered COVID-19 patients have been encouraged and approved by Food and Drug Administration (FDA) since the first months of the pandemic, due to the lack of alternative drug treatments. Although most of these clinical trials are not concluded yet, early evidence reported by completed studies demonstrated that convalescent plasma administration is a safe therapy which is able to eliminate the virus and also to improve clinical symptoms [57–60]. Notably, most of the current vaccines against SARS-CoV-2 have been designed to rise neutralizing Abs against the S protein of the virus due to their protective immunization activity [61].

In this work, the antibody response of healed and vaccinated patients was considered to search for any possible correlation between the gene/protein profile of NK cells and Abs development after virus infection or vaccine administration. Among the 6 considered patients, 1 from the *Healed group* and 1 from the *Vaccine group* did not develop Ab response (“non-responders”), whereas “responders” individuals showed protective Ab levels. Considering the expression of the 10% most variable transcripts and of all detected ADTs in NK cells, responders and non-responders within each of the *Healed group* and the *Vaccine group* keep clustering together, without any influence by the Ab development. These finding seems to suggest that vaccination or prior infection has a bigger influence on NK gene expression than the presence or absence of antibodies in the subject.

However, differentially expressed transcripts were identified among responder and non-responder patients. In particular, pathway enrichment analysis highlighted some molecular functions that are over-represented within the DEG group, and regards (protein) kinase binding, phospholipase activator activity and lipase activator activity.

During SARS-CoV-2 infection several signaling pathways can likely be activated by the interaction of S protein of the virus and the cell surface receptor ACE2. For example, kinase function plays an important role in cancer as well as in immunological, inflammatory and infectious diseases and regulation of kinase associated signaling pathway is considering an important clinical target in many pathological conditions [62]. Based on that, FDA-approved kinase inhibitors have been used as chemotherapeutic agents in cancer and as drugs with antiviral activity against a variety of viruses, including MERS-CoV and SARS-CoV-1 [63]. Kinases are thought to be involved also in the transmission of SARS-CoV-2, being related to pneumonia-like symptoms, inflammation, and fibrosis [63]. Considering this, the over-representation of the molecular function of (protein) kinase binding related to Abs development appears to be particularly intriguing, since it may suggest that NK cells are induced to express factors which regulate/inhibit kinase activity in order to contrast viral infection.

Regarding the over-representation of phospholipase activator activity pathway, it is interesting to point out that phospholipase A2 activity was proven to be significantly increased into the plasma of COVID-19 recovered patients [64]. Thus, our results may be consistent with a regulation of genes involved in this function, induced by the viral infection or vaccine administration.

Similarly, also the lipase activator activity pathway resulted to be enriched in responders individuals. Interestingly, lipase was demonstrated to be one of the enzymes whose levels are altered upon SARS-CoV‐2 infection [65, 66], with increased lipase activity registered into the blood of COVID-19 patients without acute pancreatitis [67] and worse clinical outcomes associated to elevated lipase levels [68]. Related to this, lipases inhibitors showed to suppress the replication of SARS-CoV-2 and to mitigate severe lung lesions in SARS-CoV-2-infected hamsters [69]. Our findings seem to be in line with the mentioned studies, confirming an involvement of lipase activity regulation following SARS-CoV-2 infection. In general, investigating the modification of specific cell signaling pathways related to virus pathogenesis may help to discover molecular targets for developing new therapeutic options.

Overall, these enrichment pathway results should be interpreted with caution, as the chosen threshold may not be highly conservative and the number of analysed genes is relatively limited. However, despite these limitations, these findings might still provide some insights into the transcriptional differences between NK cells from responders and subsets from non-responders individuals.

## Conclusions

In conclusion, this study detected differential expression of NK cell markers in relation to SARS-CoV-2 infection and vaccine administration, revealing a more activated NK cell phenotype in vaccinated patients *versus* recovered individuals.

Limitations of the study need to be acknowledged, firstly regarding the number of patients per group. However, although the study collected 9 blood samples, the analysis was performed on a total of ~ 270,000 cells (~ 30,000 cells/sample), which can be considered a significant number compared to previous single-cell investigation for exploratory studies about COVID-19 disease understanding [25]. Moreover, differently from previous studies, enrichment of the NK cell population was performed before single-cell analysis, with a specific selection of the immune cells of interest and not broad investigation on the whole mononuclear cell fraction. Another improvement of the work may regard the increase of the number of cell surface markers which can be analysed to better profile NK cell subsets based on the regulation of protein expression.

As future developments of the research, it would be interesting to investigate with broader case studies the evolution over time of gene/protein regulation of NK cell subsets and markers, as well as the influence exerted by concomitant chronic conditions/pathologies.

More widely, the present study contributes to the current knowledge about the application of a targeted single-cell multi-omics methodology to investigate the cellular mechanisms of COVID-19 disease, specifically focusing on NK cell population which mediates the innate immune response to the virus. By defining gene/protein markers of NK cell activation and providing preliminary evidence of cell signaling pathway induction, this pilot study paved a way for further elucidation on the role of NK cells in contributing to the immune regulation induced by SARS-CoV-2 infection and vaccine administration, also in the perspective of identifying novel targets for COVID-19 therapy.

## Electronic supplementary material

Below is the link to the electronic supplementary material.

Supplementary Material 1

Supplementary Material 2

## Data Availability

Due to the sensitive nature of the patient data involved in this study, the datasets generated and analyzed during the current study are not publicly available. However, the data can be made available upon reasonable request to the Corresponding Authors, in accordance with institutional and ethical guidelines governing patient confidentiality and data protection.
