# Supplementary material for: Innate immune response in COVID-19: single-cell multi-omics profile of NK lymphocytes in a clinical case series
Source: Cell Commun Signal. 2024 Oct 15;22:496. doi: 10.1186/s12964-024-01867-5 (PMC11476714; doi:10.1186/s12964-024-01867-5)
Supplement: Supplementary file 1 — Supplementary Material 1 [file 12964_2024_1867_MOESM1_ESM.docx]

**Additional file 1**

**Methods**

**Multi-omics analysis of NK cell samples by BD Rhapsody™**

All primary antibodies used for flow-cytometry assessment of NK cells before the analysis on the Rhapsody system were purchased by Becton Dickinson (BD) or BioLegend (San Diego, CA, USA) and are listed in Table A1.

|  | **Conjugated fluorochrome** | | | | | | | |
| --- | --- | --- | --- | --- | --- | --- | --- | --- |
|  | **FITC** | **PE** | **PerCP-Cy5.5** | **PE-Cy7** | **APC** | **APC-H7** | **BV421** | **BV480** |
| **Superscreening T-B-NK-Monocyte panel** | | | | | | | | |
| *Anti-human primary antibody* | CD3 | CD143 | CD14 | CD56 | CD16 | CD45 | CD19 + CD4 | CD8 |
| *Cat #* | 345763 | 557928 | 562692 | 335826 | 561248 | 560178 | 562440 + 566907 | 566121 |
| *Clone* | SK7 | BB9 | MΦP9 | NCAM16.2 | 3G8 | 2D1 | HIB19 + SK3 | RPA-T8 |
| **Monocyte-specific panel** | | | | | | | | |
| *Anti-human primary antibody* | HLA-DR | CCR2 | CD14 | CD11c | CD16 | CD45 | CD36 | CD64 |
| *Cat #* | 555811 | 566653 | 562692 | 561356 | 561248 | 560178 | 744766 | 746765 |
| *Clone* | G46-6 | LS132.1D9 | MΦP9 | B-Ly6 | 3G8 | 2D1 | CLBIVC7 | MD22 |
| **NK cell-specific panel** | | | | | | | | |
| *Anti-human primary antibody* | CD14 + CD19 | Siglec-7 | CD57 | CD56 | CD16 | CD3 | NKG2C | NKG2A |
| *Cat #* | 345784 + 555412 | 558372 | 393312 | 335826 | 561248 | 560176 | 748169 | 747923 |
| *Clone* | MΦP9 + HIB19 | F023-420 | [QA17A04](https://www.biolegend.com/en-us/search-results?Clone=QA17A04) | NCAM16.2 | 3G8 | SK7 | 134591 | 131411 |

**Table A1.** Primary Antibodies used for flow-cytometry assessment of NX cell populations before multi-omics analysis.

**Results**

**Enrichment of NK population from patient peripheral blood**

After NK cells enrichment by immunomagnetic separation, flow cytometry investigation allowed to characterize the immunophenotype of isolated populations before proceeding with Rhapsody analysis. Figure A1 shows the gating strategy to verify the enrichment of cell populations with phenotype CD3^-^/CD16^+^/CD56^+^.


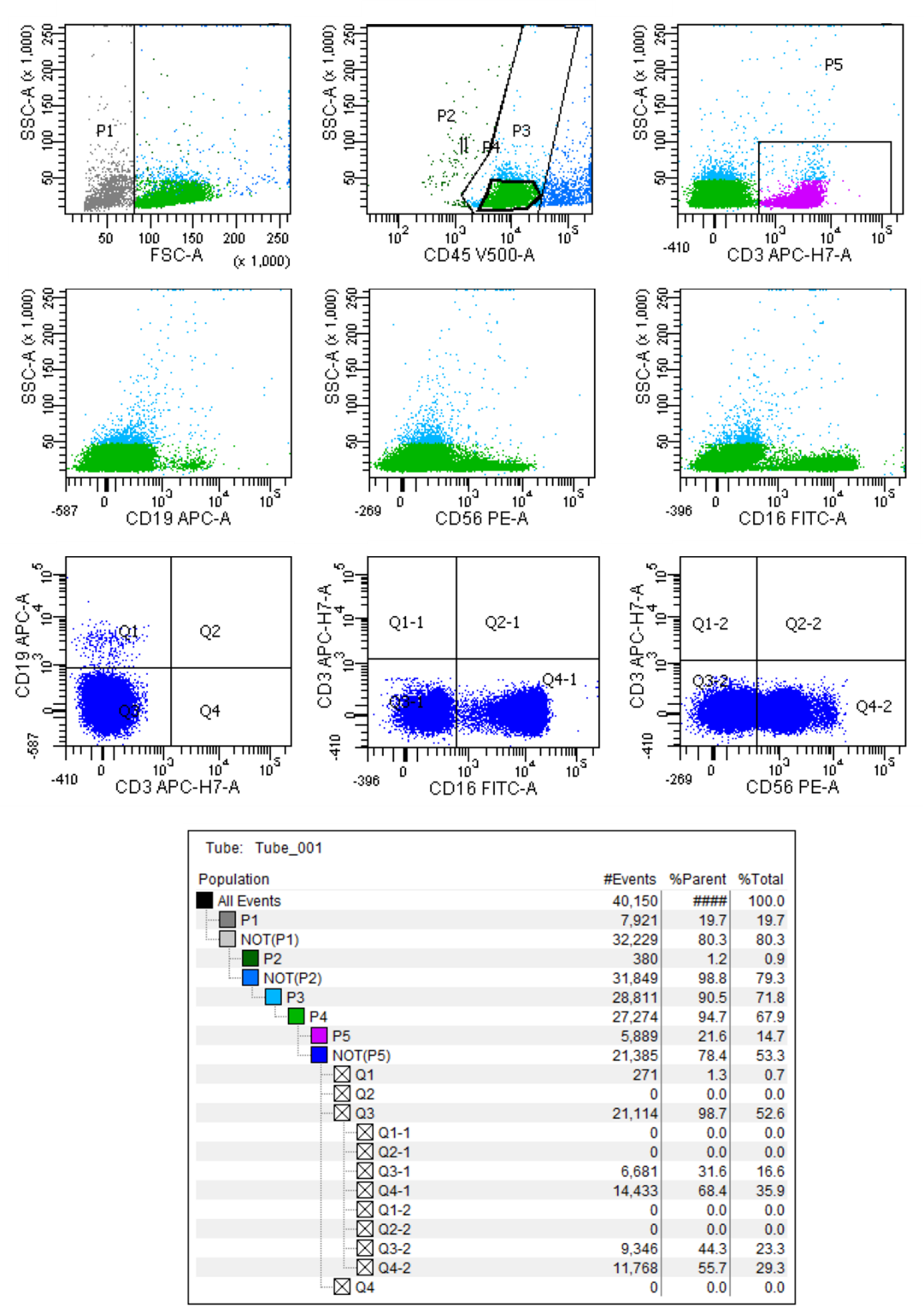


**Figure A1.** **NK cell enrichment.** Flow cytometry analysis of NK cell populations enriched by patients’ blood.

**Quality control and filtering**

The total number of cells retained per group and per sample after QC is shown in Figure A2 A, B.

The average numbers of retained mRNA transcripts and mRNA molecules per cell were comparable between groups both for mRNA (Figure A2 C) and for Antibody Derived Tag (ADT) (Figure A2 D).


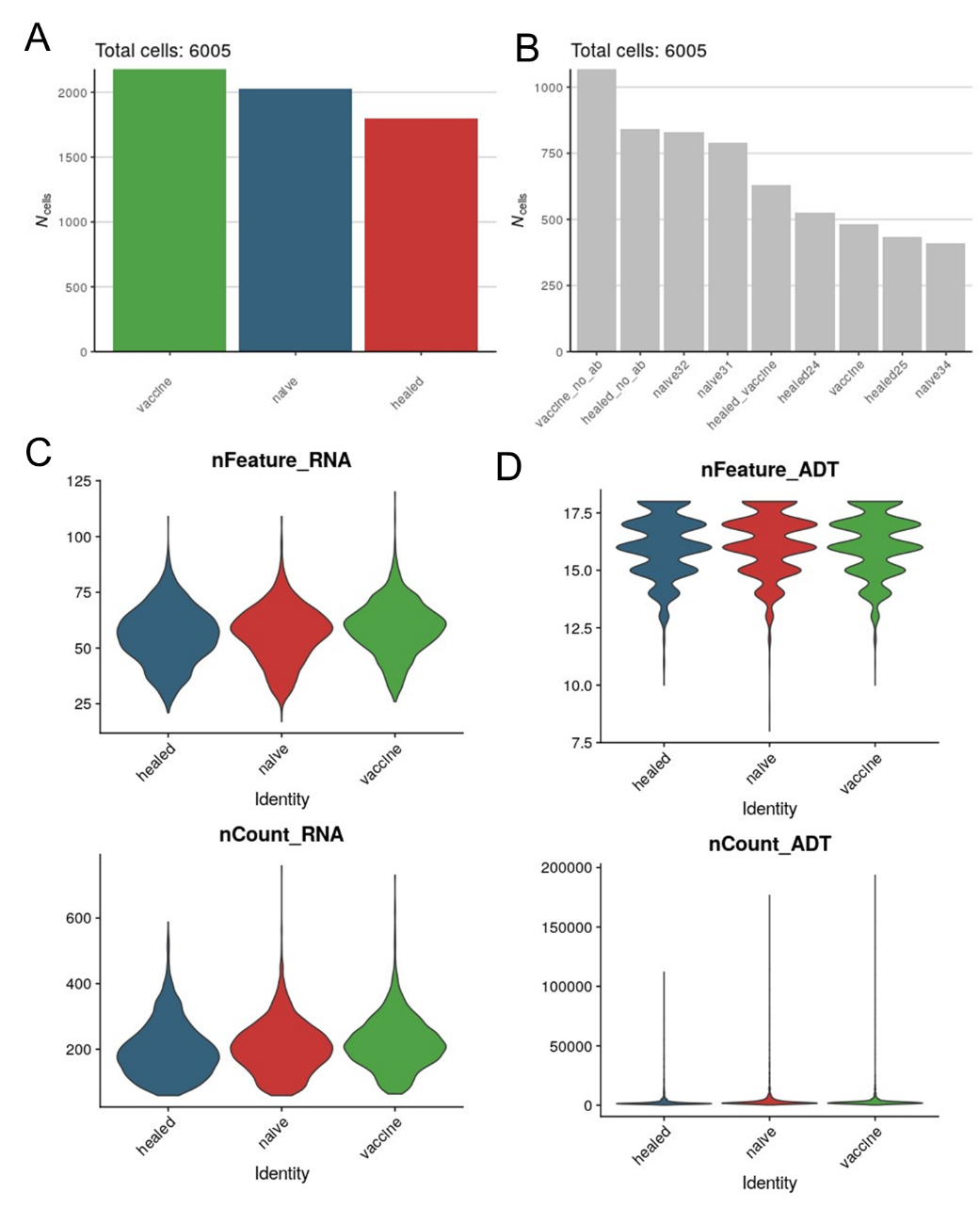


**Figure A2.** **Quality control analysis.** Total number of cells retained per group (A) and per sample (B). Average number of transcripts and molecules per group for mRNA data (C) and ADT data (D).
